# Analysis of Prevalence and Trends in the Biopsy-Proven Native Kidney Diseases in Iranian Population: A 12-year Survey from a Referral Center

**DOI:** 10.30699/IJP.2023.2000827.3104

**Published:** 2023-06-20

**Authors:** Elham Farahani, Fatemeh Nili, Mehran Moghimian, Issa Jahanzad, Farzaneh-Sadat Minoo, Alireza Abdollahi, Samaneh Salarvand

**Affiliations:** 1 *Department of Radiology, Loghman Hakim Hospital, Shahid Beheshti University of Medical Sciences, Tehran, Iran *; 2 *Department of Pathology, Imam Khomeini Hospital Complex, Tehran University of Medical Sciences, Tehran, Iran*; 3 *Department of Urology, Hasheminejad Kidney Center, Iran University of Medical Sciences, Tehran, Iran *; 4 *Nephrology Research Center, Tehran University of Medical Science, Tehran, Iran*; 5 *Center of Excellence in Nephrology, Tehran University of Medical Science, Tehran, Iran*

**Keywords:** Biopsy, Kidney disease, Prevalence

## Abstract

**Background & Objective:**

The prevalence of glomerular diseases, as the leading cause of chronic kidney disease, is increasing. Renal biopsy is still the gold standard for diagnosis of the most kidney disorders. Data on prevalence of the biopsy-proven kidney diseases in Iran is limited and none of the previously reported studies used electron microscopic (EM) evaluation for the diagnosis. This study was conducted to analyze the prevalence of biopsy-proven kidney diseases in a referral center in Iran.

**Methods:**

The reports of kidney biopsy samples from 2006 to 2018 referred to a pathology center, affiliated with Tehran University of Medical Sciences were reviewed. The prevalence of different disorders was assessed based on the clinical presentation in 3 age categories, including childhood, adulthood, and elderly.

**Results:**

Among 3455 samples, 2975 were analyzed after excluding transplant-related specimens, suboptimal specimens, and those with uncertain diagnoses. Nephrotic syndrome (NS) (39%) was the most common cause of biopsy followed by subnephrotic proteinuria (18%), hematuria in association with proteinuria (15%), renal failure (9%), isolated hematuria (6%), lupus (4%) and the other non-specific manifestations such as hypertetion or malaise (each one less than 2%). The most common diagnoses included membranous nephropathy (MGN) (17.9%), focal segmental glomerulosclerosis (FSGS) (15.9%), lupus nephritis (LN) (13.7%), minimal histopathological findings (unsampled FSGS versus Minimal Change Disease, 12.1%), Immunoglobulin-A (IgA) nephropathy (6.5%) and Alport syndrome (6.1%). MGN was the most frequent disease before 2013, but FSGS became more frequent after that.

**Conclusion:**

NS and proteinuria were the most indications for kidney biopsy. Although MGN was the most common disease, the prevalence of FSGS has been increasing in recent years and making it the most common disease after 2013. LN and IgA nephropathy are the most common causes of secondary and primary GN presenting with proteinuria and hematuria, respectively.

## Introduction

Chronic kidney disease (CKD) is a global health problem with a high mortality and morbidity burden and economic costs (1-4). The incidence of CKD is increasing worldwide (5). The number of patients needing renal replacement therapies is expected to increase up to 60% by 2030 (5). In Iran, the overall prevalence of chronic kidney disease is about 15.4%, which is higher than the global CKD prevalence (6). Although hypertension and diabetes mellitus (DM) are the most common causes of CKD in the world and Iran, the rate of glomerulonephritis is increasing globally (5, 7). 

In recent years, several noninvasive methods, including “OMICS” techniques, have been developed for the identification of early stages of kidney diseases (8). However, the impact of these biomarkers on patient management and outcome should be validated. 

Renal biopsy is still the gold standard for diagnosis, treatment approach, and predicting outcome in patients with kidney diseases (5, 8, 9). There is a scarcity of large-scale data about the prevalence of biopsy-proven kidney diseases in Iran. The distribution pattern and incidence of kidney diseases, especially glomerulonephritis have changed over time (10). Therefore, this study was conducted to analyze the prevalence of biopsy-proven kidney diseases in a referral center in Iran. All the samples were evaluated, and the diagnoses were confirmed by electron microscopy. To the best of our knowledge, this is the largest study reported from Iran.

## Material and Methods

Pathology reports of kidney biopsy samples of the patients who were referred to the Department of Surgical Pathology at Imam Khomeini Hospital, Tehran, Iran from 2006 to 2018 were reviewed. Demographic data, clinical presentation, and final diagnosis were obtained from patient documents. All native kidney biopsy samples were included in our study. Allograft and donor kidney biopsies and those with incomplete clinical data or uncertain pathologic diagnosis were excluded. All of the samples were evaluated based on the routine process for the evaluation of kidney biopsies, including light microscopy (LM), Immunofluorescence (IF), and electron microscopy (EM). The study was approved by the Ethics Committee of our university (IR.TUMS.IKHC.REC.1396.2543).

The prevalence of different disorders was assessed in 3 age categories, including childhood (<15 years old), adulthood (16-63 years old), and elderly (>64 years old). The frequency of diagnoses according to the clinical presentation (nephrotic syndrome, subnephrotic proteinuria, nephritic syndrome, hematuria, renal failure, and hypertension) was also evaluated. For evaluation of the trends in the prevalence of most common diagnoses, we compared the annual prevalence of MGN and FSGS. Collected data were analyzed using SPSS 20.1 (SPSS Inc., Chicago, Ill., USA). Continuous variables were presented using mean and standard deviation (SD) while frequency and percentage were used to present categorical variables. Independent-Sample-T and k^2 ^tests were applied to compare numerical and categorical variables. A P-value less than 0.05 was considered statistically significant. 

## Results

**Fig. 1:**
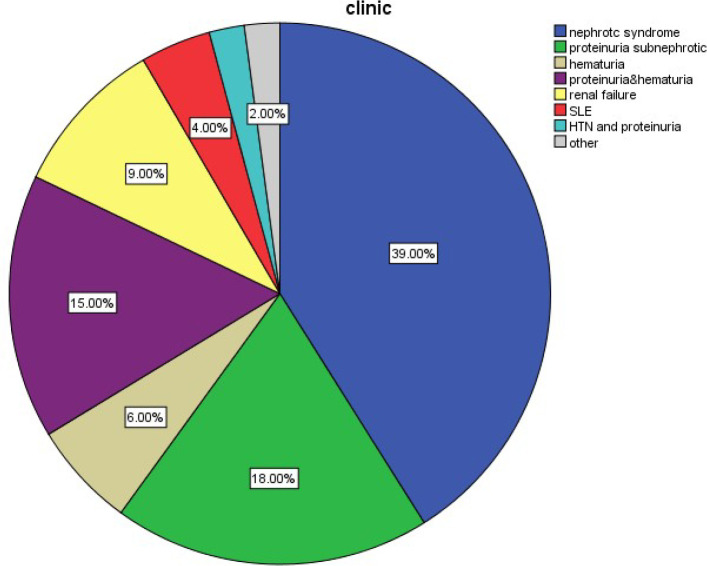
Frequency of the clinical findings in patients who underwent renal biopsy

**Fig. 2:**
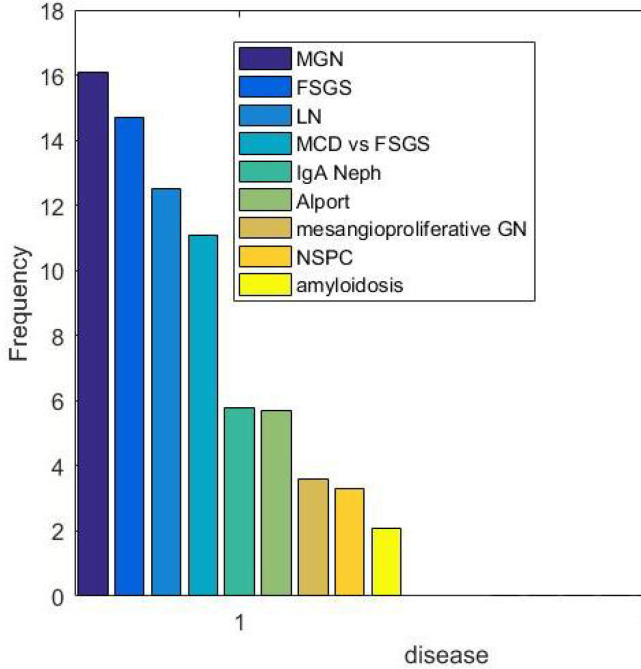
Frequency of native kidney diseases on the biopsy samples. MGN: Membranous nephropathy, FSGS: focal and segmental Glomerulosclerosis, LN: lupus Nephritis, MCD: Minimal Change disease, IgA Neph: IgA Nephropathy, NSPC: No Significant Pathologic Change

**Fig. 3:**
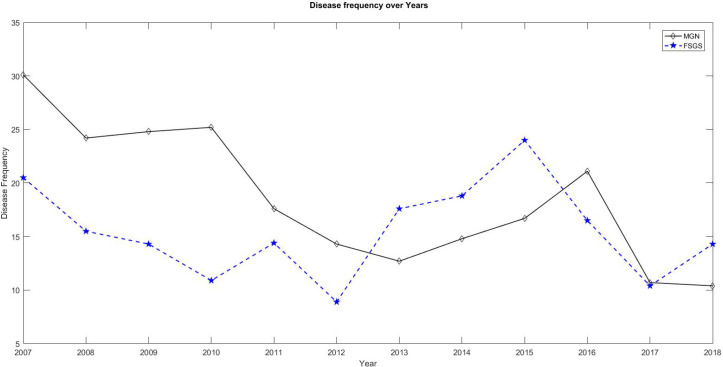
Frequency of MGN in comparison with FSGS over time

A total of 3455 samples were evaluated by LM, IF, and EM, from 2006 to 2018 in our center. Two hundred forty-four samples were kidney transplant biopsies, which were excluded from the analysis. Two hundred thirty-six samples were also excluded due to uncertain pathology results. Among the remaining 2975 cases, 51.6% were male and 48.3% of them were female. The mean age of the patients was 27.4 years old (ranging from 4 months to 86 years old). The majority (58.5%) of the cases were between 16 and 63 years old, 36.9% were children (<15 years old) and 4.6% were elderly (>64 years old). The findings of this study revealed that 5.5% of the patients had a family history of kidney diseases. As shown in Figure 1, nephrotic syndrome (39%) was the most common cause of renal biopsy followed by subnephrotic proteinuria (18%), hematuria in association with proteinuria (15%), renal failure (9%), isolated hematuria (6%), Systemic Lupus Erythematosus (SLE) (4%) and the other non-specific manifestations such as hypertetion or malaise (each one less than 2%). The frequency of the different pathologic diagnoses is shown in Figure 2. Membranous glomerulopathy (MGN) (17.9%), FSGS (15.9%), lupus nephritis (13.7%), Minimal histopathological findings (unsampled FSGS versus MCD, 12.1%), IgA nephropathy (6.5%) and Alport syndrome (6.1%) were the most common diagnoses. Rare diseases with less than 2% frequency were merged into the “others” category. As shown in Figure 3, MGN was the most common disease before 2013, but FSGS became the most frequent disease thereafter.

**Fig. 4:**
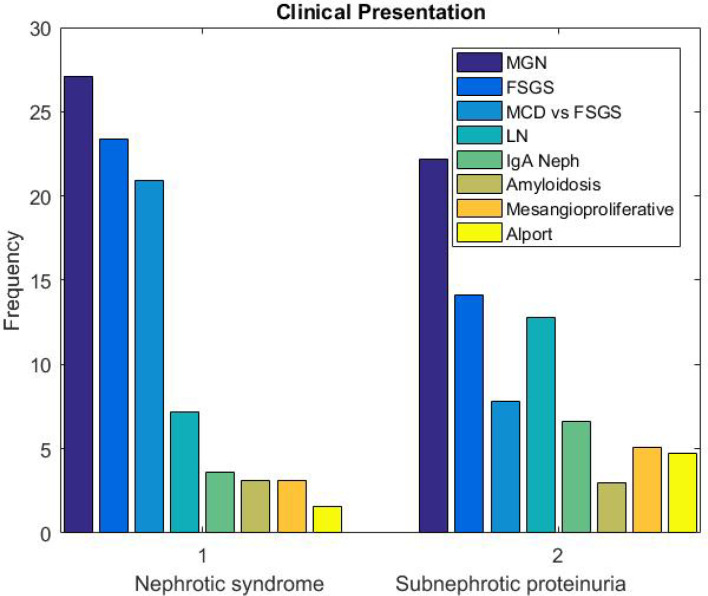
Frequency of kidney diseases in patients with clinical presentation of Nephrotic syndrome and subnephrotic proteinuria

**Fig. 5:**
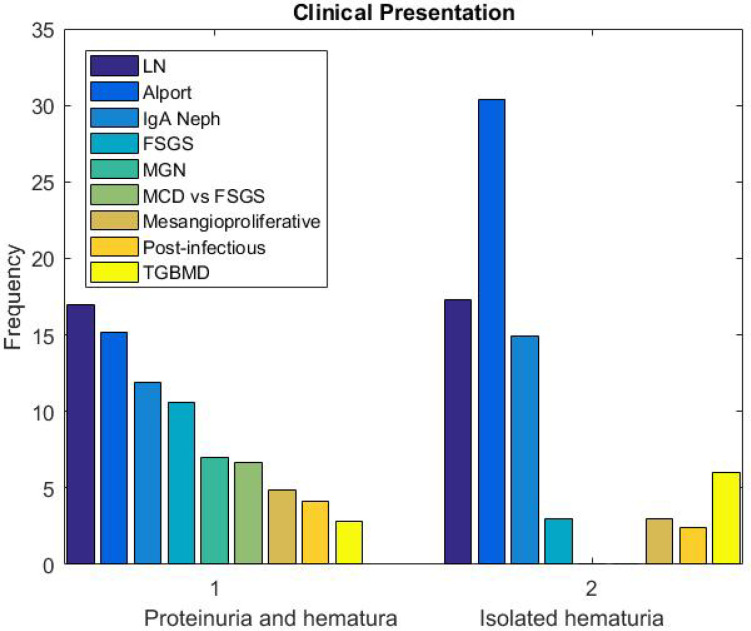
Frequency of kidney diseases in patients with clinical presentation of hematuria, proteinuria, and isolated hematuria

The prevalence of kidney diseases was more significant in males compared to females (*P*>0.05). But lupus nephritis (LN), MGN, Diabetic Nephropathy (DN) and Thin GBM Disease (TGBMD) were significantly more common among females. frequency of the pathologic diagnoses based on clinical presentation are shown in Figures 4 and 5. MGN, FSGS and MCD were the most common diagnoses in patients with nephrotic syndrome and subnephrotic proteinuria. In those with proteinuria and hematuria with or without other clinical symptoms of nephritic syndrome, LN (17%), Alport (15.2%), IgA nephropathy (11.9%) and FSGS (10.6%) were more frequently diagnosed. Alport (30.4%), LN (17.3%), IgA nephropathy (14.9%) and Thin GBM disease (TGBMD, 6%) were the most frequent diagnoses in patients with isolated hematuria. Proteinuria and HTN were most commonly seen in patients with FSGS (19.4%), MGN (13.4%), DN (9%) and CKD (7.5%). 

 The majority (84.2%) of the systematic lupus erythematosus (SLE) cases were in class III or IV while class V and II were diagnosed in 5 and 4.2% of the SLE cases, respectively. Sclerosing GN was present in 2.5% of the cases and 1.7% showed minimal histopathological findings.

Table 1 shows frequency of the kidney diseases based on clinical presentations among children, adults, and elderlies.

**Table 1:** Frequency of kidney diseases based of clinical presentations in children, adults, and elderly people

Clinic	Age classes	Diagnosis	Frequency(percentage)
Proteinuria	<15 years	MCD versus FSGS	27.5
		FSGS	22.4
	16-63 years	MGN	32.8
		FSGS	17.7
		LUPUS NEPHRITIS	10.4
	>=64 years	MGN	41.4
		AMYLOIDOSIS	18.6
		FSGS	12.9
Hematuria	<15 years	ALPORT	32.3
		LUPUS NEPHRITIS	18.5
		IgA NEPHROPATY	13.1
		INCONCLUSIVE	11.5
	16-64years	IgA NEPHROPATY	17.4
		LUPUS NEPHRITIS	15.2
		NSPC	13.0
	>=64 years	MGN	40.0
		IgA NEPHROPATY	20.0
		Proliferative GN	20.0
Proteinuria and Hematuria	<15 years	LUPUS NEPHRITIS	19.8
		ALPORT	17.3
	16-63 years	LUPUS NEPHRITIS	14.8
		IgA NEPHROPATHY	14.8
		FSGS	10.7
		MGN	10.1
	>=64 years	FSGS	27.3
		MGN	18.2

## Discussion

Despite the development of non-invasive diagnostic methods for kidney diseases, OMICS analyses, renal biopsy, and pathology assessment are still considered the gold standard methods for diagnosing kidney diseases (8). For standard pathologic assessments of each sample, light microscopy, immunofluorescence, and electron microscopic evaluation have been recommended. But in Iran as in many other regions, electron microscopy is not available in the majority of the centers. This study presents the largest data in which all of the cases, including adult and pediatric samples, were evaluated by electron microscopy. 

The most frequent indications for kidney biopsy were nephrotic syndrome, subnephrotic range of proteinuria, hematuria, proteinuria, isolated hematuria, lupus, and renal failure, respectively. These findings were in line with the findings of many other previous studies (11-13).

In our study, men were more frequently affected by most of the kidney diseases (male/female ratio=1.27) except for lupus nephritis, TGBMD, and MGN, in which there was female predilection (14). This finding was in accordance with other studies (14). Although MGN was found to be more common among men according to previous data (male/female ratio of about 2:1) (15, 16), however, it is better to differentiate a primary from secondary membranous nephropathy to evaluate the exact epidemiology of primary MGN, which was not performed for all cases in our study. In children, the most common indication for renal biopsy was nephrotic syndrome and minimal histopathological abnormality was the most prevalent pathologic finding. In this group, MCD and unsampled FSGS were the differential diagnoses. 

The mean age of our patients at the time of biopsy was less than most other studies because our study included children and adults. Furthermore, these findings might be influenced by the difference in the threshold for renal biopsy. 

In both adults and elderlies who presented with nephrotic syndrome or subnephrotic proteinuria, MGN was the most prevalent diagnosis followed by FSGS. In some other studies, the prevalence of FSGS was more than MGN among the African American population (17, 18). But in Caucasians, MGN has been reported to be more common (17, 18). In another study, primary MGN was reported as the most common condition in whites, followed by Asians, blacks, and Hispanics (16). In a large recent study performed in China, IgA nephropathy and MGN were shown to be the most frequent diagnoses (10). In one study on the Korean population, IgA nephropathy was demonstrated to be the most common diagnosis followed by MCD and MGN (19). The results of studies on the Iranian population are also variable. In the studies by Ossareh, Jafari *et al., *and Naini 1407, 130 and 469 patients were analyzed, respectively. They found MGN as the most common diagnosis (12, 20, 21). Daneshpajouhnejad and Mohammadhoseiniakbari *et al.* evaluated 1054 and 393 cases respectively and reported that FSGS was the most common disease (13, 22). All of these previous studies had a smaller sample size compared to our study. Moreover, they were from different regions of Iran, which may influence the results. On the other hand, in none of the previous studies, electron microscopy was available for definite diagnosis. However, electron microscopy is necessary for the evaluation of foot process effacement and differentia-ting primary forms of FSGS from the secondary causes of glomerular sclerosis. In our study, we found a number of patients (24 cases) with the diagnosis of FSGS based on light microscopy who were diagnosed with Alport syndrome after electron microscopic evaluation. Finally, most of the previous studies have shown a trend in FSGS diagnosis over the past years (9, 10, 17, 18, 23). This finding was in line with the findings of our study that indicated FSGS as the most common disease after 2013 till now. Among the Iranian studies, Daneshpajouhnejad *et al.* found FSGS as the most common disease in 2016 compared to the results of other studies that were published before 2014 (13). It should be noted that the true prevalence of FSGS may also be underestimated because of its nature, including the involvement of focal glomeruli with segmental pattern which could be unsampled in small biopsies and be misdiagnosed as MCD (8). Therefore, we analyzed this group of cases as a separate group "MCD versus FSGS". The diagnosis of FSGS was confirmed at the follow-up of most adult patients, who presented with subnephrotic proteinuria, or associated hematuria and HTN.

LN was the most common diagnosis in patients with proteinuria in association with hematuria with or without a complete nephritic pattern. Lupus nephritis, which usually presents as nephritic syndrome, was the most common cause of secondary GN in many other studies. Among our LN cases, which were reported based on the 2003 ISN/RPS classification, class IV and III (+/-V) were the most frequent diagnoses (84.2%). This finding was concordant with the findings of other studies, which reported class IV LN as the most common diagnosis (24, 25). 

IgA nephropathy and Alport syndrome, are the most common forms of primary glomerulonephritis presenting as proteinuria and hematuria. As mentioned previously, IgA nephropathy is the most common cause of hematuria worldwide and the most common GN in many populations. The findings of this study revealed that Alport syndrome was the most frequent disease in patients with isolated hematuria, especially in pediatric patients. The exact prevalence of Alport syndrome is not well-known, but it is estimated to be 1 per 50000 live births in the United States and 1 per 100000 in Europe (26). The incidence of Alport syndrome in our study seems to be higher than in some other countries (13-14 new cases per year). Two reasons could be considered for this difference: 1) limited access to EM and underestimation of the diagnosis in other studies, and 2) overestimation of the diagnosis in our center as we received many samples of clinically suspected Alport syndrome from different parts of Iran. 

In the elderly population, the most common cause of nephrotic syndrome and subnephrotic proteinuria was MGN followed by amyloidosis and FSGS, which was in line with the findings of the study by Perkowska-Ptasinska *et al.* in Poland (27). They found MGN, FSGS, and amyloidosis as the most common diseases. Therefore, amyloidosis should be considered in any patient older than 64 years old with proteinuria and thus additional work-up should be performed for such patients.

The fact which should be mentioned here is that the difference in the threshold of biopsy indication in renal involvement in most diseases (i.e. diabetic nephropathy, lupus nephritis, or urinary abnormalities) as well as geographical, racial, and sex differences can affect the frequency of the pathologic diagnosis of renal diseases (28-30). Larger multicenter studies can aid in more relevant results.

## Conclusion

Nephrotic syndrome and proteinuria are the most common indications for kidney biopsy in Iranian patients. Although MGN is the most common diagnosis, it has been replaced by FSGS in recent years (from 2013 onwards). LN and IgA nephropathy are the most common causes of secondary and primary GN presenting with proteinuria and hematuria, respectively.

## Conflict of Interest

There are no conflicting interests.
